# The effects of chemotherapy on resting energy expenditure, body composition, and cancer-related fatigue in women with breast cancer: a prospective cohort study

**DOI:** 10.1186/s40170-023-00322-2

**Published:** 2023-11-09

**Authors:** Timia Van Soom, Wiebren Tjalma, Konstantinos Papadimitriou, Nick Gebruers, Eric van Breda

**Affiliations:** 1 Department of Rehabilitation Sciences & Physiotherapy, Research Group MOVANT, Multi-Disciplinary Metabolic Research Unit (M2RUN), Faculty of Medicine and Health Sciences, University of Antwerp, Universiteitsplein 1, Antwerp, 2610 Belgium; 2 Multidisciplinary Breast Clinic, Antwerp University Hospital (UZA), Wilrijkstraat 10, 2650 Edegem, Belgium; 3 Multidisciplinary Edema Clinic, Antwerp University Hospital (UZA), Wilrijkstraat 10, 2650 Edegem, Belgium; 4 Department of Medicine, Faculty of Medicine and Health Sciences, University of Antwerp, Universiteitsplein 1, 2610 Antwerp, Belgium; 5 General Hospital Rivierenland, Kasteelstraat 23, 2880 Bornem, Belgium

## Abstract

**Background:**

Breast cancer (BC) is the most prevalent tumor in women. Improvements in treatment led to declined mortality, resulting in more survivors living with cancer- or therapy-induced comorbidities. In this study, we investigated the impact of neoplasia and chemotherapy on resting energy expenditure (REE) and body composition, in relation to cancer-related fatigue. Inflammatory parameters were checked as possible explanation for changes in REE.

**Methods:**

Fifty-six women participated: 20 women with BC and 36 healthy controls. Patients were assessed at baseline (T0) and follow-up (T1) after 12 weeks of chemotherapy. Controls were measured once. REE was assessed with indirect calorimetry: body composition (body weight, fat mass, fat-free mass) by air plethysmography. The multidimensional fatigue index (MFI-20) was used to analyze fatigue. Baseline measurements of patients were compared to results of the healthy controls with the independent-samples *T*-test. The paired-samples *T*-test investigated the effects of chemotherapy from T0 to T1. A Pearson correlation analysis was conducted between REE, body composition, and fatigue and between REE, body composition, and inflammatory parameters. A linear regression analysis was fitted to estimate the contribution of the significantly correlated parameters. The measured REE at T0 and T1 was compared to the predicted REE to analyze the clinical use of the latter.

**Results:**

At baseline, patients with BC had significantly higher REE in the absence of differences in body composition. From baseline to T1, REE and body weight did not change. In contrast, fat-free mass declined significantly with concordant increase in fat mass. Fatigue deteriorated significantly. C-reactive protein at baseline predicted the change in energy expenditure. Predicted REE significantly underestimated measured REE.

**Conclusions:**

Women with BC have higher REE in the tumor-bearing state compared to healthy controls. Chemotherapy does not affect REE but alters body composition. Predictive equations are invalid in the BC population. Results of our study can be used to implement personalized nutritional interventions to support energy expenditure and body composition and minimize long-term comorbidities.

## Introduction

Globally, breast cancer (BC) is the most prevalent neoplasm in women [1, 2]. Despite the growing incidence, mortality is decreasing as a result of better screening methods and advancements in therapy, adding to the growing number of survivors [3, 4]. As a result, the consequences of increasing survival rates lead to long-lasting comorbidities, such as diabetes, cardiovascular disease, and fatigue [4, 5].

Women with BC who receive adjuvant chemotherapy (CT) are metabolically compromised. This altered metabolic profile in combination with drugs and lifestyle changes may lead to alterations in resting energy expenditure (REE) [4, 5]. Several studies have shown that REE is increased in cancer patients, which may be one of the causative factors for, at least, physical fatigue as it might ease the onset of malnutrition. In the malnourished state, nutritional intake does not meet the increased energy expenditure, with negative effects on physical performance and functional capacity [6, 7].

Different methods have been developed to determine REE, amongst them numerous equations that predict REE (pREE) [8]. In clinical practice, the Harris-Benedict equation (HB_Eq_) is often applied. The HB_Eq_ estimates REE based on anthropometric and demographic factors such as body height (BH), body weight (BW), sex, and age [3]. Only in healthy subjects, it has been shown that 85% of the general population has a measured REE (mREE) within 10% of predicted REE by the HB_Eq_ [9]. Although predictive equations are valid in overall healthy individuals without any form of intervention or lifestyle change, they are subjected to over- or underestimation in patients or during periods of interventions.

One of the major challenges in cancer patients is to differentiate between tumor-induced changes in REE or and/or chemotherapy-induced changes in REE [10]. This is important because chemotherapeutic agents have toxic side effects that could affect energy expenditure and add to the tumor induced changes in REE and may increase feelings of fatigue additionally.

To investigate the energy expenditure and possible coinciding effects on cancer-related fatigue (CRF) and quality of life (QoL), we conducted a longitudinal prospective cohort study on women with BC, all receiving CT, and investigated absolute and relative levels of energy expenditure, body composition (BW, FM, and FFM), and patient-reported CRF. We also examined inflammatory parameters, as possible explanations for metabolic changes. A better understanding of the potential side effects of CT may provide clinicians with more insight and help to counteract the possible debilitant effects of CT.

## Materials and methods

This approved study (Belgian registration: B300201837317) was conducted at the Multidisciplinary Metabolic Research Unit (M^2^RUN) of Movant Research Group (University of Antwerp) in cooperation with the Multidisciplinary Breast Clinic of the University Hospital of Antwerp (*Belgium*).

### Design

This prospective cohort study investigated the effects of a 12-week treatment period with paclitaxel chemotherapy (CT; 1 × /week, 12 weeks) on energy expenditure, body composition, and fatigue. More specifically, we investigated absolute resting energy expenditure (mREE), mREE in relation to fat-free mass (relative mREE; mREE/FFM), metabolic state (mREE/pREE [11, 12]), and body composition (BW, FFM, and FM) in patients with BC before (T0) and after (T1) treatment (12 weeks; 1 administration/week). We also examined the validity of the often-used HB_Eq_ to predict REE (pREE) in the BC population before and after the treatment. Finally, the effects of a 12-week CT on CRF, and of blood parameters (total neutrophile count (tNC); total lymphocyte count tTLC), neutrophile-to-lymphocyte ratio (NEU/LYM), C-reactive protein (CRP)), or tumor size at T0 on the outcome parameters related to energy expenditure and body composition were investigated.

### Participants

Women diagnosed with primary BC were recruited from the Multidisciplinary Breast Clinic of the University Hospital of Antwerp (October 2020-September 2022). Participants were included when they were > 18 years of age, diagnosed with BC, and registered for neoadjuvant treatment with paclitaxel chemotherapy (CT; 12 administrations 1 × /week) with no prior treatment (e.g., surgery). Since the presence of the tumor in BC patients might influence our primary outcome parameters, a healthy control group was recruited for comparison at the start of the study. The healthy controls were recruited after advertising on social platforms (Facebook, Instagram, Twitter). Eligible subjects were contacted by telephone and provided with baseline appointment at the laboratory. In- and exclusion criteria of both the patient and healthy control group can be found in Table 1.

**Table 1:** In- and exclusion criteria of the study population

Group	Inclusion criteria	Exclusion criteria
**Patients with breast cancer**	• Women • > 18 years • First diagnosis of breast cancer • Primary breast cancer • Registered for neoadjuvant chemotherapy (paclitaxel)	• Men with breast cancer • < 18 years • Breast cancer as secondary tumor (metastasis) • Prior treatment for breast cancer (e.g., surgery)
**Healthy controls**	• Women • Age: 40–65 years • General good health as follows: The absence of metabolic diseases (hyper-/hypothyroidism, burn wounds, liver diseases) and < 2 metabolic dysfunctions (hypertension, hypertriglyceridemia, hypercholesterolemia, insulin resistance, increase in blood glucose) • No common cold or flu < 2 weeks ago • No surgery < 1 month ago • No current use of pharmaceuticals • No cancer experienced in the past	• Men • Age: < 40 years; > 65 years • The presence of metabolic diseases, > 2 metabolic dysfunctions • Common cold or flu < 2 weeks ago • Surgery < 1 month ago • Current use of pharmaceuticals • Cancer experienced in the past

### Procedures and outcome measures

All patients with BC were invited twice to the research facility. A baseline assessment at T0 of REE, body composition, and fatigue was executed before the first administration of CT, while the follow-up measurement (T1) was planned after 12 weeks of CT program.

As a comparison, healthy volunteers were assessed only once at T0, to determine whether or not patients with BC had a tumor-induced difference in REE and scored differently in the domains of MFI-20. Indirect calorimetry (IC) was used to measure mREE, while air displacement plethysmography (BOD POD) was used for analyzing body composition (BW, FFM, FM). Fatigue was assessed by the multidimensional fatigue inventory (MFI-20).

All assessments were executed in the morning (7h00am–11h30am). Participants were asked to be in fasted state (minimum 8 h without eating). Drinking water was allowed ad libitum until 2 h prior to the measurement. All subjects were asked to refrain from heavy exercise 24 h before the measurement. Each appointment lasted 1.5 h. Written informed consent was provided by each participant.

#### Characteristics of the study sample

The following data were collected or calculated from measurements in the laboratory, the patient’s medical record, or from an anamnesis (healthy controls): age (years); BW (kg); BH (cm); body mass index (BMI); weight status was determined according to the following reference values: underweight: BMI < 18.5, normal weight: BMI = 18.5–24.9, and overweight/obese: BMI ≥ 25.0; pREE (calculated by $HBeq=447.593+\left(9.247 x BW\left(kg\right)\right)+\left(3.098 x BH\left(cm\right)\right)-\left(4.330 x age\left(y\right)\right)$); the metabolic state was defined by the % difference between mREE and pREE using the following reference values (hypermetabolism (mREE/pREE >  + 10%), hypometabolism (mREE/pREE <  − 10%), normometabolism (mREE/pREE =  ± 10%)); blood parameters (tLC, tNC, NEU/LYM, CRP); and tumor size (mm).

#### Indirect calorimetry

All IC measurements were done with an open-circuit diluted flow calorimeter (*Omnical IV*, *Maastricht Instruments*, the Netherlands). Calibration of the device was performed automatically every 30 min with span gas (18% O_2_ and 0.8% CO_2_) and nitrogen gas (100%). Validation of the system by methanol combustion was performed weekly [13].

The measurements were executed in a respiratory room (14 m^3^). Participants were instructed to lay in a semi-inclined position while staying awake. Minimal activity (reading, desk work, listening to music) was allowed. The room served as reservoir collecting $\dot{\mathrm{V}}$ O_2_ and $\dot{\mathrm{V}}$ CO_2_. The measurement lasted 60 min, and data were provided every minute. Results of the last 50 min were used. $\dot{\mathrm{V}}$ O_2_ and $\dot{\mathrm{V}}$ CO_2_ were converted to REE values (Weir, 1949). Data were collected as kcal/min and recalculated to kcal/24 h (absolute mREE) to determine relative mREE. Results on $\dot{\mathrm{V}}$ O_2_ and $\dot{\mathrm{V}}$ CO_2_ can be found as supplementary material (Table 9 in Appendix).

#### BOD POD

FM and FFM were assessed by air-displacement plethysmography (BOD POD, *COSMED*, USA) [14, 15]. The device was calibrated prior to each analysis, as prescribed by the manufacturer. The relative contribution of FM to body weight (FM%) was calculated by the SIRI equation: $FM\%=\left(\frac{495}{body density}\right)-450$. Next, the relative contribution of FFM to body weight (FFM%), total amount of FM (kg), and FFM (kg) was derived.

#### Multidimensional fatigue inventory

The MFI is a 20-item self-report instrument to measure five dimensions of fatigue: General fatigue, physical fatigue, mental fatigue, reduced motivation, and reduced activity. Each dimension is covered by 4 items (questions). Participants were asked to score the items on a 5-point Likert scale. Each dimension received a score on 20, with higher scores indicating more fatigue on that specific domain. The grading system was analyzed with the validated scoring tool (MFI-20 scoring tool; Dutch version [16]).

### Statistical analysis

Quantitative variables are expressed as mean ± SD. All data were checked for normality (Kolmogorov–Smirnov, QQ-plot, and histogram). Demographic characteristics include the following: age, BH, BW, obesity status (according to BMI), and metabolic state (based on mREE/pREE: hypermetabolism, hypometabolism, normometabolism). Blood- or tumor-related parameters include the following: tNC, tLC, NEU/LYM, CRP, and tumor size. Metabolic parameters include the following: absolute mREE, relative mREE, and mREE/pREE; parameters related to body composition include the following: FM, FFM, and BW; and domains of fatigue include the following: general fatigue, physical fatigue, mental fatigue, reduced motivation, and reduced activity.

The independent samples *T*-test analyzed the difference in means of the demographic variables between BC and healthy controls at T0. The difference in means of measures of energy expenditure and body composition from T0 to T1 in patients with BC was analyzed by the paired samples *T*-test. The independent samples *T*-test analyzed the difference between the metabolic parameters and body composition parameters of BC and controls at T0. The paired samples *T*-test investigated the difference in means between absolute mREE and pREE, as calculated by the HB_eq_, to determine its validity in patients with BC at T0 and T1. The paired samples *T*-test was also used to analyze the difference in the five domains of fatigue in patients with BC from T0 to T1. In addition, the independent samples *T*-test was used to determine the difference in fatigue between patients with BC and healthy controls at baseline.

The correlation between the metabolic parameters, body composition, and fatigue, at T0 and T1, is expressed by the Pearson correlation coefficient (r). In addition, the correlation between the change from T0 to T1 (delta) of the metabolic parameters and body composition, blood parameters, and tumor size is also examined by the Pearson correlation coefficient. Next, a linear hierarchical regression model was fitted to estimate the contribution of the significantly correlated parameters to the change in aspects of energy metabolism and body composition. The individual contribution is expressed by the *R*^2^ change. All statistical tests are executed two-sided (significance: *α* < 0.05). Statistical analyses were performed with SPSS software (SPSS v29, IBM Business Analytics, New York, USA).

## Results

### Participants

Fifty-six women participated in the study: 20 women diagnosed with BC, and 36 healthy women were enrolled in the control group. Six women with BC were lost in follow-up: two due to aggravation of their medical condition, and four were unable to attend the measurements at follow-up due to time constraints, leaving a total of 14 BC patients in the study.

Characteristics of the study population and comparison of means between groups can be found in Table 2. At baseline, age, BW, BH, and BMI did not differ significantly between patients and controls. The ratio between mREE and pREE was statistically significant (*BC*: 16.4 ± 12.3 vs. controls: 2.5 ± 7.0; *p* < 0.001).

**Table 2:** Comparison between groups at baseline (T0)

Parameter	Cancer (*n* = 20) Mean ± SD	Controls (*n* = 36) Mean ± SD	*p*-value
**Age**	55.30 ± 11.0	52.9 ± 5.4	0.188
**BW**	70.1 ± 5.9	70.4 ± 13.8	0.906
**BH**	168.5 ± 6.0	167.4 ± 6.4	0.546
**BMI**	24.9 ± 3.4	25.1 ± 4.3	0.904
*18.0 (n%)*	0 (0%)	0 (0%)	**-**
**18.0–24.9 (n%)**	9 (45.0%)	(52.8%)	**-**
** ≥ 25.0 (n%)**	11 (55.0%)	17 (47.2%)	**-**
**Metabolic state (mREE/pREE)**	16.4 ± 12.3	2.5 ± 7.0	< 0.001*
**Hyper (n%)**	6 (50.0%)	7 (19.4%)	**-**
**Normo (n%)**	7 (42.9%)	28 (77.8%)	**-**
**Hypo (n%)**	1 (7.1%)	1 (2.8%)	**-**

Legend: *n*, number of participants; *mREE*, measured resting energy expenditure; *pREE*, predicted resting energy expenditure; *FFM*, fat-free mass; *BW*, body weight; *FM*, fat mass; *BH*, body height; *BMI*, body mass index; hyper, hypermetabolism; hypo, hypometabolism; normo, normometabolism; significance

*p < 0.05

### Resting energy expenditure and body composition

#### Patients with BC vs. controls at baseline

At T0, absolute mREE was significantly higher (± 11%) in patients with BC compared to controls (*BC*: 1596.8 ± 212.1 vs. controls: 1421.9 + 153.1; *p* < 0.001). The same was true for relative mREE (mREE/FFM) (± 10%) (*BC*: 34.4 ± 4.8 vs. controls: 31.0 ± 3.0; *p* < 0.001). The ratio between mREE and pREE also differed significantly (*BC*: 15.8 ± 9.2 vs. controls: 2.5 ± 7.0; *p* < 0.001). No significant differences in body composition parameters (BW_kg_, FFM_kg_, FFM_%_, FM_kg_, FM_%_) were found between patients and controls at T0.

### Effects of chemotherapy in BC: from baseline to follow-up

From T0 to T1, absolute mREE, relative mREE, and mREE/pREE did not change significantly in patients with BC. Body weight and FM_kg_ were also not significantly different. However, FFM_kg_ (T0: 46.6 ± 4.6 vs. T1: 45.1 ± 3.9; *p* = 0.006) and FFM_%_ (T0: 66.8 ± 6.6 vs. 63.6 ± 3.9; *p* = 0.028) were significantly lower in BC at T1 compared to T0, while FM_%_ (T0: 33.2 ± 6.6 vs. T1: 39.6 ± 8.6; *p* = 0.027) was significantly higher at T1 (Table 3).

**Table 3:** Comparison of energy expenditure and body composition between patients with breast cancer and controls

Parameter	Cancer (*n* = 20) Mean ± SD	Controls (*n* = 36) Mean ± SD	*p*-value
mREE	1596.8 ± 212.1	1421.9 ± 153.1	< 0.001*
mREE/FFM	34.4 ± 4.8	31.0 ± 3.0	< 0.001*
mREE/pREE	15.8 ± 9.2	2.5 ± 7.0	< 0.001*
BW	70.1 ± 5.9	70.4 ± 13.8	0.906
FFM_kg_	46.6 ± 4.6	46.1 ± 5.4	0.812
FFM_%_	66.8 ± 6.6	65.1 ± 11.8	0.966
FM_kg_	24.2 ± 7.2	26.0 ± 13.6	0.915
FM_%_	33.2 ± 6.6	33.3 ± 8.5	0.497
Effects of 12 weeks of chemotherapy energy expenditure and body composition in patients with breast cancer			
Parameter	Baseline (T0; *n* = 14) Mean ± SD	Follow-up (T1; *n* = 14) Mean ± SD	*p*-value
mREE	1596.8 ± 212.1	1524.0 ± 230.9	0.174
mREE/FFM	34.4 ± 4.8	33.8 ± 4.2	0.628
mREE/pREE	15.8 ± 9.2	9.8 ± 13.8	0.084
BW	70.1 ± 5.9	71.7 ± 5.2	0.204
FFM_kg_	46.6 ± 4.6	45.1 ± 3.9	0.006*
FFM_%_	66.8 ± 6.6	63.6 ± 3.9	0.028*
FM_kg_	24.2 ± 7.2	25.9 ± 4.0	0.208
FM_%_	33.2 ± 6.6	39.6 ± 8.6	0.027*

Legend: *n*, number of participants; *mREE*, measured resting energy expenditure; *pREE*, predicted resting energy expenditure; *FFM*, fat-free mass; *BW*, body weight; *FM*, fat mass; significance

*p < 0.05

### Comparison between measured and predicted energy expenditure

At baseline, the HB_Eq_ significantly underestimated mREE in patients with BC and healthy controls. For patients with BC, pREE was ± 13% lower compared to mREE (*pREE*: 1384.4 ± 121.7 vs. *mREE*: 1596.8 ± 212.1; *p* < 0.001). At T1, pREE underestimated mREE with ± 8.4% in patients (*pREE*: 1395.8 ± 81.4 vs. *mREE*: 1524.0 ± 230.9; *p* = 0.019) (Table 4).

**Table 4:** Comparison between indirect calorimetry (mREE) and predictive Harris-Benedict equation (pREE)

Time of measurement	mREE Mean ± SD	pREE Mean ± SD	*p*-value
Cancer			
Baseline	1596.8 ± 212.1	1384.4 ± 121.7	< 0.001* (underestim. ± 13%)
Follow-up	1524.0 ± 230.9	1395.8 ± 81.4	0.019* (underestim. ± 8.4%)
Control			
Baseline	1421.9 ± 153.1	1388.1 ± 133.9	0.048* (underestim. ± 2.4%)

Legend: *n*, number of participants; *mREE*, measured resting energy expenditure; *pREE*, predicted resting energy expenditure; significance

*p < 0.05

### Effects of chemotherapy on reported fatigue

#### Patients with BC vs. controls at baseline

At baseline, patients with BC were significantly less active in comparison with control subjects (decreased activity; *BC*: 11.6 ± 4.0 vs. controls: 7.4 ± 3.0; *p* < 0.001). Higher levels of general fatigue, physical fatigue, decreased motivation, and mental fatigue were also seen in patients, although differences were not significant (Table 5).

**Table 5:** Comparison of fatigue between patients with breast cancer and controls at baseline (T0)

Parameter	Cancer (*n* = 20) Mean ± SD	Control (*n* = 36) Mean ± SD	*p*-value
General fatigue	10.9 ± 3.4	9.3 ± 3.8	0.093
Physical fatigue	10.1 ± 4.6	9.2 ± 3.7	0.419
Decreased activity	11.6 ± 4.0	7.4 ± 3.0	< 0.001*
Decreased motivation	7.9 ± 3.2	6.6 ± 2.5	0.161
Mental fatigue	8.7 ± 4.8	8.6 ± 4.4	0.546
Effects of 12 weeks of chemotherapy on fatigue in patients with breast cancer			
Parameter	Baseline (T0) (*n* = 14) Mean ± SD	Follow-up (T1) (*n* = 14) Mean ± SD	*p*-value
General fatigue	10.9 ± 3.4	14.5 ± 3.3	0.297
Physical fatigue	10.1 ± 4.6	14.9 ± 3.1	0.027*
Decreased activity	11.6 ± 4.0	12.9 ± 3.5	0.235
Decreased motivation	7.9 ± 3.2	9.4 ± 3.9	0.002*
Mental fatigue	8.7 ± 4.8	8.9 ± 4.1	0.004*

Legend: *n*, number of participants; significance

*p < 0.05

### Effects of chemotherapy in BC: from baseline to follow-up

Patients with BC experience significantly more physical fatigue (T0: 10.1 ± 4.6 vs. T1: 14.9 ± 3.1; *p* = 0.025) and mental fatigue (T0: 8.7 ± 4.8 vs. T1: 8.9 ± 4.1; *p* = 0.002) and report significantly decreased motivation (T0: 7.9 ± 3.2 vs. T1: 9.4 ± 3.9; *p* = 0.002) during chemotherapy treatment (Table 5). Despite slightly higher levels, general fatigue as well as decreased activity did not change over the course of the treatment period with CT.

#### Correlation analysis between aspects of energy expenditure, body composition, and fatigue in patients with BC

At T0, absolute mREE correlated moderately but significantly with BW, relative mREE, and mREE/pREE (*r*: 0.524, *p* = 0.021; *r*: 0.530, *p* = 0.019 and *r*: 0.710, *p* < 0.001, respectively) and correlated low with FFM_kg_ (*r*: 0.455, *p* = 0.049). Nonsignificant low correlations were found between absolute mREE and all other parameters of body composition. Furthermore, relative mREE displayed a significantly negative moderate correlation with FFM_kg_ (*r*: − 0.509, *p* = 0.026) but a significant strong positive correlation with mREE/pREE (*r*: 0.799, *p* < 0.001). The ratio mREE-pREE was strongly and significantly correlated with absolute and relative mREE, while the correlation with all other parameters was low and non-significant. General fatigue, physical fatigue, decreased motivation, and mental fatigue were not significantly correlated with body composition, absolute mREE, relative mREE, or mREE/pREE. For decreased activity, a significant low-positive correlation was found with FFM_kg_ (*r*: 0.465, *p* = 0.039) and a moderate negative correlation with relative mREE (*r*: − 0.576, *p* = 0.010). All other parameters were low but nonsignificantly correlated (Table 6).

**Table 6:** Correlation analysis between energy expenditure, body composition, and fatigue at T0

Parameter	mREE	mREE/FFM	mREE/pREE	General fatigue	Physical fatigue	Decreased activity	Decreased motivation	Mental fatigue
BW	0.524*	− 0.125	− 0.135	0.187	0.285	0.102	0.136	0.115
FFM_kg_	0.455*	− 0.509*	− 0.083	0.181	0.304	0.465*	0.338	0.026
FFM_%_	− 0.177	− 0.367	0.103	− 0.071	− 0.110	0.325	0.197	− 0.146
FM_kg_	0.430	0.176	− 0.065	0.025	0.129	− 0.252	− 0.096	0.062
FM_%_	0.177	0.367	− 0.103	0.071	0.110	− 0.325	− 0.197	0.146
mREE	1	0.530*	0.710*	− 0.134	0.103	− 0.158	0.068	− 0.093
mREE/FFM	0.530*	1	0.779*	− 0.283	− 0.173	− 0.576*	− 0.269	− 0.115
mREE/pREE	0.710*	0.799*	1	− 0.341	− 0.146	− 0.350	− 0.082	− 0.270
Correlation analysis between energy expenditure, body composition, and fatigue at T1								
Parameter	mREE	mREE/FFM	mREE/pREE	General fatigue	Physical fatigue	Decreased activity	Decreased motivation	Mental fatigue
BW	0.832*	0.582*	0.641*	− 0.208	− 0.176	− 0.103	− 0.428	0.065
FFM_kg_	0.523	− 0.034	0.267	− 0.221	− 0.058	0.011	− 0.069	− 0.067
FFM_%_	− 0.417	− 0.748*	− 0.541*	− 0.110	0.118	0.234	0.521	− 0.068
FM_kg_	0.765*	0.805*	0.754*	− 0.017	− 0.165	− 0.249	− 0.608*	0.010
FM_%_	0.218	0.212	0.233	0.252	0.066	0.548*	− 0.287	0.163
mREE	1	0.832*	0.922*	− 0.300	− 0.348	− 0.248	− 0.414	− 0.217
mREE/FFM	0.832*	1	0.905*	− 0.200	− 0.356	− 0.268	− 0.431	− 0.178
mREE/pREE	0.922*	0.905*	1	− 0.212	− 0.275	− 0.261	− 0.270	− 0.287

Legend: *mREE*, measured resting energy expenditure; *pREE*, predicted resting energy expenditure; *FFM*, fat-free mass; *BW*, body weight; *FM*, fat mass

*High correlation

At T1, absolute mREE, relative mREE, mREE/pREE, and FM_kg_ were significantly and strongly correlated with BW (*r*: 0.832, *p* < 0.001, *r*: 0.832, *p* < 0.001, *r*: 0.905, *p* < 0.001, and *r*: 0.765, *p* = 0.001, respectively). Strong significant correlations were also seen between relative mREE and FM_kg_ (*r*: 0.805, *p* < 0.001), absolute mREE (*r*: 0.832, *p* < 0.001), and mREE/pREE (*r*: 0.922, *p* < 0.001). BW correlated moderately with relative mREE (*r*: 0.582, *p* = 0.029). A significant negative correlation was found between relative mREE and FFM_%_ (*r*: − 0.748, *p* = 0.002). A strong significant positive correlation was seen for mREE/pREE with FM_kg_ (*r*: 0.754, *p* = 0.002). FFM_%_ was significantly moderate but negatively correlated with mREE/pREE (*r*: − 0.541, *p* = 0.046), while BW correlated moderate and positively with mREE/pREE (*r*: 0.641, *p* = 0.014).

General fatigue, physical fatigue, and mental fatigue did not correlate with any of the energy expenditure or body composition parameters. A moderate correlation was found between decreased activity and FM_%_ (*r*: 0.548, *p* = 0.035) and between decreased motivation and FM_kg_ (*r*: − 0.608, *p* = 0.021). All other parameters correlated low to moderately, but non-significantly, within the domains of fatigue (Table 6).

#### Correlation and regression analysis between blood parameters, tumor size, and the change in energy expenditure and body composition in patients with BC

No correlation was found between tLC at T0 and changes in energy expenditure, BW, FFM_kg_, and FM_kg_. Interestingly, tNC at T0 had a significantly strong positive correlation with the change in BW (*r*: 0.768; *p* = 0.001) as did NEU/LYM at T0 (*r*: 0.643; *p* = 0.013). A moderate negative correlation was found for CRP at T0 and the change in absolute mREE (*r*: − 0.656; *p* = 0.015), relative mREE (*r*: − 0.733; *p* = 0.004), and mREE/pREE (*r*: − 0.708, *p* = 0.007). A moderate negative correlation was found between tumor size at T0 and the change in absolute mREE (*r*: − 0.553; *p* = 0.050), relative mREE (− 0.630, *p* = 0.021), and mREE/pREE (*r*: − 0.614, *p* = 0.026) (Table 7).

**Table 7:** Correlation analysis between the change in energy expenditure, body composition, and confounding factors

Parameter	ΔmREE	ΔmREE/FFM	ΔmREE/pREE	ΔBW	ΔFFM_kg_	ΔFFM_%_	ΔFM_kg_	ΔFM_%_
T0_lymphocytes	0.242	0.306	0.227	0.087	− 0.279	− 0.324	0.413	0.095
T0_neutrophiles	0.453	0.454	0.238	0.768*	− 0.165	− 0.434	0.281	− 0.032
T0_Neu/Lym	0.175	0.144	− 0.012	0.643*	0.052	− 0.098	− 0.046	− 0.095
T0_CRP	− 0.656*	− 0.733*	− 0.708*	0.009	0.029	− 0.023	0.087	− 0.002
T0_tumor size	− 0.553*	− 0.630*	− 0.614*	0.094	0.485	0.123	− 0.031	0.511

Legend: *n*, number of participants; *Δ*change from baseline to follow-up; *mREE*, measured resting energy expenditure; *pREE*, predicted resting energy expenditure; *FFM*, fat-free mass; *BW*, body weight; *FM*, fat mass; *Neu/Lym*, level of neutrophiles/level of lymphocytes; *CRP*, level of C-reactive proteins

CRP at T0 was the single significant predictor for the change for all aspects of energy expenditure. For absolute mREE, the *R*^2^ was 0.656 with an *R*^2^ change of 0.430 (F(1.11) = 8.311, *p* = 0.015). For relative mREE, the *R*^2^ was 0.733 with an *R*^2^ change of 0.537 (F(1.11) = 12.739, *p* = 0.004). For mREE/pREE, the *R*^2^ was 0.708 with an *R*^2^ change of 0.501 (F(1.11) = 11.027, *p* = 0.007) tNC at T0 which significantly predicted the change in BW with *R*^2^ was 0.768 and *R*^2^ change of 0.590 (F(1.12) = 17.240, *p* = 0.001) (Table 8).

**Table 8:** Linear regression analysis: confounding factors of blood parameters and tumor size for the change in aspects of energy metabolism and body weight

Parameter	*R*^2^	*R*^2^ change	*p*-value
**ΔmREE**			
**Model** ***T0_CRP***	0.656	0.430	0.015*
**ΔmREE/FFM**			
**Model** ***T0_CRP***	0.733	0.537	0.004*
**ΔmREE/pREE**			
**Model** ***T0_CRP***	0.708	0.501	0.007*
**ΔBW**			
**Model** ***T0_****n****eutrophiles***	0.768	0.590	0.001*

Legend: *n*, number of participants; *Δ*change from baseline to follow-up; *T0*, baseline; *mREE*, measured resting energy expenditure; *pREE*, predicted resting energy expenditure; *FFM*, fat-free mass; *CRP*, level of C-reactive proteins; significance

*p < 0.05

## Discussion

This is one of the few studies that investigated resting energy expenditure in breast cancer patients before and after chemotherapeutic treatment (CT). The main and novel findings of this study are that patients with BC have a significantly higher resting energy expenditure prior the start of treatment with paclitaxel compared to healthy controls, and that treatment with paclitaxel had no additional effects on resting energy expenditure. We also found that the metabolic aberrations at baseline were present in the absence of significant differences in body composition between patients and controls. Furthermore, we found differences in body composition, e.g., body weight increased nonsignificantly, but FFM_kg,%_ showed a significant decline in combination with a significant increase in FM_%_.

### Effects of breast cancer on energy expenditure and body composition

At present, we have no decisive explanation for the significant higher REE at baseline in comparison with healthy controls. However, tumor burden, determined by a.o. tumor location and size, affects energy metabolism subsequently [8]. Our results on tumor size confirm this finding, as a larger tumor size was negatively related to the change in energy expenditure throughout the treatment. It makes sense that, despite their relative small size, a tumor is a very active metabolic tissue, characterized by high rates of glycolysis and lactate metabolism (Cori cycle) [17, 18]. Upregulation of these metabolic pathways in cancer increases energy expenditure and may lead to a negative energy balance which consequently affects body weight and body composition [8]. Based on our findings and the literature, we suggest that the increased REE in BC patients is caused by the tumor itself. Our results (absolute mREE in patients with BC was 11% higher compared to healthy controls) support the findings of Nguyen et al. [19], who found substantial evidence towards an 8–9% elevation of REE in the tumor-bearing state compared to an overall healthy population. REE is the sum of the energy expenditure needed for metabolic activities of various organs and tissues during resting conditions, except for sleep and arousal, without any physical activity [20, 21]. In healthy adults, fat-free mass (FFM) is the largest predictor of REE as it consists mainly of, amongst other, skeletal muscle tissue that has a high metabolic activity [22, 23]. Since skeletal muscle mass makes up approximately 35–40% of total BW, changes in REE are often accompanied by changes in FFM and reflect the compensatory physiologic strategies to meet the energy demands of the individual metabolic processes [20]. Tüccar et al. [8] suggested that adjusting for FFM is necessary to correctly interpret REE [8]. Here, we show that the relative mREE in BC was also significantly higher (10%) compared to the control group. Our results substantiate the idea that the tumor in BC patients elevates energy expenditure significantly [19]. Depending on type, stage, and location [24], a tumor is estimated to consume an extra 100–1400 kcal/day as indicated by Friesen et al. [18], which has a substantial effect on REE [8, 18]. In theory, an added metabolic demand of 300 kcal/day is 25% of the energy expenditure of a patient with an REE of 1200 kcal/day but only 15% for someone with an REE of 2000 kcal/day [8]. Consequently, large variations are observed in literature regarding the additional metabolic demand amongst patients in different cancer types, including BC [19]. Besides the energetic impact of tumor itself, inflammation also leads to an increase in REE [25]. Interestingly, Purcell et al. (2020) found a positive relationship between inflammation, defined by the level of CRP, and REE in stages III/IV colorectal patients [23]. In our study, CRP at baseline correlated significantly negative with the change in energy expenditure. As inflammation was present in all our patients (*CRP*: 9.09 ± 19.6 mg/L), we hypothesize that the higher REE is induced by inflammatory pathways as well, as previously shown by Madzima et al. [26] in similar patients as in our study.

An important aspect that needs to be addressed is that our patient population was significantly less active at baseline in comparison to the control group, while all other fatigue levels of the MFI-20 subdomains were higher in the BC group, although nonsignificantly. We have no clear explanation for the findings of a decreased level of physical activity without concomitant increases in any of the fatigue subdomains of the MFI-20 at baseline. However, although not further investigated in current study, we can hypothesize that mental fatigue negatively influences the level of physical activity as it affects exercise decision-making [27]. Moreover, cancer-induced metabolic changes (e.g., upregulation of glycolysis and Cori-cycle) often are often seen to be related with a plethora of symptoms, of which fatigue is one of the most commonly reported [28]. Further research is necessary to unravel the onset and mechanisms of fatigue and possible correlations with abnormal metabolic patterns in cancer patients.

### Effects of chemotherapy on resting energy expenditure and body composition

After 12 weeks of treatment, a nonsignificant decrease (− 73 kcal/day) in resting energy expenditure was seen in BC patients. This is in line with the findings of Harvie et al. [29] who observed a decline in measured REE (− 93 kcal/day) 3 months after chemotherapy treatment. Such findings have been confirmed by some investigators [3, 30–32] but are opposed to others who reported increases in energy expenditure during chemotherapy treatment [33]. Body composition on the other hand changed significantly from T0 to T1 in patients with BC. In our study, a significant loss of FFM (FFM_kg_: − 1.5 kg; FFM_%_: − 3.2%) and significant gain in FM_%_ (+ 6.4%; FM_kg_: + 1.7 kg), without a concomitant altered BW (+ 1.6 kg), could be observed. Our findings on BW confirm the result of a meta-analysis by van de Berg et al. [34], who reported, despite a large heterogeneity observed, an average increase of 2.7-kg BW in women with BC during CT. Both our findings and literature point out that weight changes and changes in body composition are predominantly characterized by an increase in FM and decrease FFM [35].

In healthy adults, the contribution of FFM to REE is fivefold greater than the contribution of FM to REE [36]. Considering this, we can assume that a significant decrease in FFM leads to concomitant decline in REE, as modeled by Wang et al. [22]. In contrast, our results demonstrate a maintained energy expenditure, despite a significant decline in FFM. A decrease in FFM during CT occurs mostly due to depletion of skeletal muscle mass resulting from increased protein breakdown. Both in the illness stage as well as during treatment with CT, proteolysis-inducing factors are secreted activating the adenosine triphosphate (ATP)-dependent proteolytic system, resulting in increased protein catabolism with consequently diminishing effects on the total amount of FFM. Since the latter is REE’s biggest determinant, a decrease in REE can be expected as well. However, in line with our findings, studies suggest that REE increases (despite loss of BW and FFM) as a result from these catabolic processes, which are often accompanied by inflammation [8, 19, 24].

Muscle atrophy during CT is associated with an increase in inflammatory cytokines, such as CRP, and related higher energy expenditure [37]. Although specific inflammatory cytokines (such as interleukin (IL)-6, IL-11, and tumor necrosis factor α (TNF-α)) were not further investigated and known for being present in tumor related inflammation [38], we hypothesize that the zero net change in REE found in our study might result from an elevated metabolic demand related to inflammation, obscuring the diminishing effects of the loss of FFM on REE [23]. This hypothesis is supported by our findings on tumor size and the level of CRP at baseline, which were significantly negative correlated with the change in energy expenditure, with CRP as sole significant predictor.

In clinical practice, measures of BW and body composition are useful outcomes to interpret results on REE at a single time point, but cannot detect inflammation and tumor burden which seem to affect energy expenditure as well [23]. This might explain the difference between predicted and measured energy expenditure. In our study, REE predicted by HB_Eq_ significantly underestimated REE measured by indirect calorimetry, confirming previous results [39]. Our findings underline the importance of a precise assessment of REE when adapting nutritional strategies, as the metabolic demand of not only body composition (FFM) but also the energetic costs of a tumor and concurrent systemic effects need to be accounted for.

This study has limitations worth discussing. First of all, the relatively small patient sample might have influenced our clinical results. Although the strength of our study is the measurement of REE in the tumor-bearing state before the start of chemotherapy, the short timespan between diagnosis and first administration impeded patient inclusion. More, patients were not screened on cancer-related cachexia before entering the study. With our inclusion criteria, however, we hope to have limited the risk of including cachectic patients, already metabolically compromised. Also, analysis of specific inflammatory cytokines (such as interleukins) could have provided more information to support our findings on inflammation. Additionally, we did not control for dietary intake. Consequentially, the results of the respiratory exchange ratio (RER; substrate oxidation) could not be used, as it is highly influenced by nutrient intake on the previous days. Future studies should include RER as well, as it provides important information on the impact of chemotherapeutic agents on nutrient utilization, which will improve patient-tailored dietary advice. Furthermore, it is possible that repeated measurements (> 2) of energy expenditure during treatment unravels a dynamic pattern which is currently outbalanced. Finally, we included only patients that received neoadjuvant paclitaxel chemotherapy; other chemotherapeutic drugs and treatment regimens might have different outcomes.

## Conclusion

Our study concludes that patients with breast cancer have significantly higher energy expenditure compared to healthy controls, and that a 12-week treatment regimen with paclitaxel chemotherapy did not alter energy expenditure, however, it had an impact on body composition. A non-significant increase in BW was seen alongside a significant decline in FFM_kg,%_ and concurrent increase in FM_%_. We hypothesize that tumor burden and inflammation mask the effects of diminished FFM on energy expenditure, and results in elevated REE levels. We promote the importance of precise assessment of REE by indirect calorimetry when adapting nutritional strategies, as the HB_Eq_ significantly underestimates the true energy needs in patients. Furthermore, a decrease in the level of activity was present in patients compared to the control group, with no difference in FFM. All other domains of fatigue were higher in the patient group and deteriorated significantly after 12 weeks of chemotherapy. Our results can found the basis for implementing supportive exercise and dietary rehabilitation during treatment with CT as it will promote the conservation/amelioration of body composition profiles, positively influencing both prognosis and cancer related QoL.

## Data Availability

The datasets generated and analyzed during the current study are available from the corresponding author on reasonable request.

## Appendix

**Table 9:** Comparison of VO_2_ and VCO_2_

Parameter	Baseline Mean ± SD	Follow-up Mean ± SD	*p*-value
Patients with BC			
VO_2_	235.5 ± 30.3	218.5 ± 31.1	0.058
VCO_2_	162.9 ± 47.2	175.7 ± 41.9	0.124
Time of measurement	Cancer Mean ± SD	Control Mean ± SD	*p*-value
Baseline (T0)	*n* = 19	*n* = 36	
VO_2_	235.5 ± 30.3	205.7 ± 22.0	**< 0.001***
VCO_2_	162.9 ± 47.2	159.5 ± 18.9	0.596

Legend: *BC*, breast cancer; *VO*_*2*_, oxygen uptake; *CO*_*2*_, carbohydrate expenditure; *T0*, baseline; significance

**p* < 0.05
